# Interaction between human lung fibroblasts and T-lymphocytes prevents activation of CD4^+ ^cells

**DOI:** 10.1186/1465-9921-6-103

**Published:** 2005-09-13

**Authors:** Carlo Vancheri, Claudio Mastruzzo, Elisa Trovato-Salinaro, Elisa Gili, Debora Lo Furno, Maria P Pistorio, Massimo Caruso, Cristina La Rosa, Claudia Crimi, Marco Failla, Nunzio Crimi

**Affiliations:** 1 Department of Internal and Specialistic Medicine, Section of Respiratory Medicine, University of Catania, Catania, 95125, Italy

**Keywords:** COX-2, ICAM-1, CD3, CD28, LFA

## Abstract

**Background:**

T lymphocytes are demonstrated to play an important role in several chronic pulmonary inflammatory diseases. In this study we provide evidence that human lung fibroblasts are capable of mutually interacting with T-lymphocytes leading to functionally significant responses by T-cells and fibroblasts.

**Methods:**

Human lung fibroblast were co-cultured with PMA-ionomycin activated T-CD4 lymphocytes for 36 hours. Surface as well as intracellular proteins expression, relevant to fibroblasts and lymphocytes activation, were evaluated by means of flow cytometry and RT-PCR. Proliferative responses of T lymphocytes to concanavalin A were evaluated by the MTT assay.

**Results:**

In lung fibroblasts, activated lymphocytes promote an increase of expression of cyclooxygenase-2 and ICAM-1, expressed as mean fluorescence intensity (MFI), from 5.4 ± 0.9 and 0.7 ± 0.15 to 9.1 ± 1.5 and 38.6 ± 7.8, respectively. Fibroblasts, in turn, induce a significant reduction of transcription and protein expression of CD69, LFA-1 and CD28 in activated lymphocytes and CD3 in resting lymphocytes. In activated T lymphocytes, LFA-1, CD28 and CD69 expression was 16.6 ± 0.7, 18.9 ± 1.9 and 6.6 ± 1.3, respectively, and was significantly reduced by fibroblasts to 9.4 ± 0.7, 9.4 ± 1.4 and 3.5 ± 1.0. CD3 expression in resting lymphocytes was 11.9 ± 1.4 and was significantly reduced by fibroblasts to 6.4 ± 1.1. Intracellular cytokines, TNF-alpha and IL-10, were evaluated in T lymphocytes. Co-incubation with fibroblasts reduced the number of TNF-alpha positive lymphocytes from 54,4% ± 6.12 to 30.8 ± 2.8, while IL-10 positive cells were unaffected. Finally, co-culture with fibroblasts significantly reduced Con A proliferative response of T lymphocytes, measured as MTT absorbance, from 0.24 ± 0.02 nm to 0.16 ± 0.02 nm. Interestingly, while the activation of fibroblasts is mediated by a soluble factor, a cognate interaction ICAM-1 mediated was demonstrated to be responsible for the modulation of LFA-1, CD28 and CD69.

**Conclusion:**

Findings from this study suggest that fibroblasts play a role in the local regulation of the immune response, being able to modulate effector functions of cells recruited into sites of inflammation.

## Introduction

Interactions between immunocompetent cells, such as lymphocytes and monocytes/macrophages, and other hematopoietic cell lineages is an essential and well known feature of the immune and inflammatory response. Much less attention has been given to the possibility of direct and mutual interactions between immunocompetent cells and resident cells such as fibroblasts. To this regard we have previously shown that normal human lung fibroblasts interact with monocytes suggesting their involvement in the control of the immune and inflammatory response [1,2]. In addition, we have demonstrated that an impairment of fibroblast functions, as observed in fibrotic fibroblasts, may lead to a reduced capability of these cells to modulate monocyte activity [2]. Several data indicate that in pulmonary chronic inflammatory diseases, such as bronchial asthma and interstitial lung diseases, lymphocytes are in an immunologically activated state likely as the result of a persistent and excessive state of immune activation, possibly due to a dysregulation of the fine homeostatic balance governing the immune response [3-5]. In this context, very limited attention has been addressed to potential direct interactions between T lymphocytes and lung fibroblasts [6,7]. Recent studies have in fact provided evidence that the interaction between lymphocytes and fibroblasts might be important to the pathogenesis of chronic inflammatory diseases such as periodontitis and rheumatoid arthritis. In periodontitis, T lymphocytes are often found adjacent to gingival fibroblasts [8] whereas in the inflammed synovium, T lymphocytes and fibroblasts along with monocytes/macrophages, represent the most abundant cell populations. With regard to these disease conditions, it has been demonstrated that T cells induce the activation of both gingival and synovial fibroblasts [9,10]. In addition, it has recently been shown that stromal cells are able to affect T-cell apoptosis, contributing to the accumulation and/or removal of these cells at sites of chronic inflammation [11-13]. However, the inappropriate retention of T-cells within the tissue is unlikely to be the only mechanism leading to the switch from an acute resolving to a chronic persistent inflammatory process and it is reasonable to think that a persistent and excessive condition of immune activation of these cells may be important as well. In view of the above findings, that fibroblasts are capable of interacting with T-lymphocytes, we set out to determine whether the interaction between normal human lung fibroblasts and T-cells could lead to a functionally significant response by T-lymphocytes, influencing their state of immune activation. Our results indicate that lung fibroblasts and T-lymphocytes indeed mutually interact. Activated lymphocytes induce the expression of cyclooxygenase-2 (COX-2) and dramatically increase the expression of intercellular adhesion molecule-1 (ICAM-1) in normal human lung fibroblasts. Fibroblasts, in turn, induce a significant reduction of transcription and protein expression of CD69, considered as a marker of early T cell activation, lymphocyte function associated antigen-1 (LFA-1), CD3 and CD28, all molecules involved in T-lymphocyte activation and costimulation [14-16].

According to this phenotypic down-regulation, lymphocytes co-cultured with fibroblasts, show a significant reduction of the production of tumor necrosis factor-α (TNFα), while the production of interleukin-10 (IL-10) is not affected. This condition of reduced activation is further underlined by a reduced proliferation of lymphocytes co-cultured with fibroblasts in response to a mitogenic stimulus.

It is interesting to note that while the activation of fibroblasts is mediated by a soluble factor, a cognate interaction between ICAM-1 and LFA-1 is responsible for the modulation of LFA-1, CD28 and CD69 on T-cells.

These data confirm and expand the concept that human lung fibroblasts may actively interact with immune cells affecting a large array of functions strictly related to the control and regulation of the local immune response.

## Materials and methods

### Lung Fibroblasts

Seven primary lines of normal human adult lung fibroblasts were established by using an outgrowth from explant according to the method described by Jordana and coworkers [17]. Fibroblast lines were derived from histologically normal areas of surgical lung specimens from patients undergoing resective surgery for cancer. Their ages ranged from 52 to 61 yr. Five of six patients were men. Lung specimens were chopped into pieces of less than 1 mm^3 ^and washed once with PBS and twice with RPMI-1640 containing 10% FCS, penicillin 100 U/ml, streptomycin 100 mcg/ml, and fungizone 25 mcg/ml (supplemented RPMI) (Gibco, Paisley, UK); eight to ten pieces of washed specimens were then plated in a 100-mm polystyrene dish (Falcon, Becton Dickinson, Lincoln Park, NJ, USA) and overlaid with a coverslip held to the dish with sterile vaseline. Ten milliliters of supplemented RPMI were added and the tissue was incubated at 37°C with 5% CO_2_. The medium was changed weekly. When a monolayer of fibroblast-like cells covered the bottom of the dish, usually 5 to 6 weeks later, the explant tissue was removed, and the cells were then trypsinized for ten minutes, resuspended in 10 ml of supplemented RPMI, and plated in 100-mm tissue culture dishes. Subsequently, cells were split 1:2 at confluence, usually weekly. Aliquots of cells were frozen and stored in liquid nitrogen. In all experiments we used cell lines at a passage earlier than the tenth.

### Lymphocyte isolation procedure

Heparinized venous blood, obtained from healthy donors, was diluted 1:3 with PBS, and 40 ml were then placed on 10 ml of Lymphoprep (Axis-Shield, Oslo, Norway) for centrifugation at 1,600 rpm for 35 minutes at room temperature. Mononuclear cells were collected at the interface, washed three times and resuspended in PBS supplemented with 0.5% bovine serum albumin and 2 mM EDTA. Isolation of human CD4 lymphocytes from mononuclear cells was performed by positive selection of CD4^+ ^cells using a magnetic cell sorting system (MACS, Miltenyi Biotec, Bergisch Gladbach, Germany) according to manufacturer's instructions. Mononuclear cells were magnetically labeled with CD4 microbeads and passed through a separation column placed in the magnetic field of the MACS separator. The magnetically labeled cells were retained in the column while the unlabeled cells run through. After removal of the column from the magnetic field labeled cells, representing the enriched CD4^+ ^cell fraction, passed through the column and were collected as effluent.

### Lymphocyte-Fibroblast Co-cultures

Lymphocytes were incubated in 60 mm polystyrene dish (Falcon, Becton-Dickinson) at a concentration of 4 × 10^6 ^cells in 4 ml of supplemented RPMI in the absence or presence of 1 μg/ml of ionomycin and 10 ng/ml of PMA, plates were then incubated in a humidified atmosphere of 5% CO_2 _at 37°C. After 6 hours cells were harvested, washed three times with PBS and counted. 1 × 10^6 ^lymphocytes were then seeded on top of 0.5 × 10^6 ^fibroblasts in 6-well tissue culture plates in a final volume of 2 ml of supplemented RPMI and incubated for 36 hours. After the 36 hours of co-culture fibroblasts were adherent to the dish and maintained the typical spindle shaped aspect. Lymphocyte viability was assessed by the trypan blue exclusion method that constantly gave a >90% survival. In some experiments cells were separated by a semipermeable membrane (0.4 mcm pores) using a cell culture insert (Falcon, Becton Dickinson). In blocking experiments fibroblasts were pretreated with a blocking anti-ICAM antibody (Calbiochem Corporation, San Diego, CA, USA) for 2 hours before the addition of the lymphocytes and once again when the co-culture started.

### RNA Isolation and Reverse Transcriptase-Polymerase Chain Reaction

Total cellular RNA was extracted from cells with the guanidium isothiocyanate/acid-phenol procedure as previously described [18]. The yield and the purity of RNA was measured spectrophotometrically by absorption at 260/280 nm. Total RNA was used for the generation of cDNA. Reverse transcriptase-polymerase chain reaction (RT-PCR) was performed using the SuperScript™ First-Strand Synthesis System for RT-PCR (Invitrogen Inc., Paisley, UK), with some modifications. Briefly, 5 μg of total RNA was reverse transcribed with 50 U of RNase OUT Recombinant (Superscript™ II RT, Invitrogen). The reverse-transcribed product (cDNA) was amplified by PCR (Perkin Elmer Gene Amp PCR System 2400) in the presence of a master mix containing PCR buffer, MgCl_2 _(under optimal concentrations), 1 U Taq DNA Polymerase Recombinant (Invitrogen), 10 mM dNTPs. The following specific primer pairs were used: ICAM-1 sense 5'-GAGCTGTTTGAGAACACCTC-3' and antisense 5'-TCACACTTCACTGTCACCTC-3' giving a 367 bp PCR product; COX-2 sense 5'-TTCAAATGAGATTGTGGGAAAATTGCT-3' and antisense 5'-AGATCATCTCTGCCTGAGTATCTT-3' (305 bp product); LFA-1 sense 5'-GTCCTCTGCTGAGCTTTACA-3' and antisense 5'-ATCCTTCATCCTTCCAGCAC-3' (337 bp product); CD-28 sense 5'-AAGTTGAGAGCCAAGAGCAG-3' and antisense 5'-CCGACTATTTTTCAGTGACA-3' (304 bp product); CD-69 sense 5' CCTTCCAAGTTCCTGTCC-3' and antisense 5' CATTCCATGCTGCTGACCTC-3' (451 bp product); CD-3 sense 5' GTGTCATTCTCACTGCCTTGTTCC-3' and antisense 5'-TTCAGTGGCTGAGAAGAGTGAACC-3' (496 bp product); beta-actin sense 5'-TGACGGGGTCACCCACACTGTGCCCATCTA-3' and antisense 5'-CTAGAAGCATTGCGGTGGACGATGGAGGG-3' (661 bp product). PCR was performed for 40 cycles, using a cycling program of 94°C for 5 min, 55°C for 59 sec and 72°C for 59 sec in a thermal cycler for the amplification of ICAM-1 and COX-2, for the amplification of LFA-1 and CD-28, PCR was performed for 35 cycles, using a cycling program of 94°C for 5 min, 54°C for 59 sec and 72°C for 59 sec, while for the amplification of CD-69 and CD-3 PCR was performed for 25 and 30 cycles, using a cycling program of 94°C for 5 min, 52°C and 57°C for 59 sec and 72°C for 59 sec, respectively. Final extension was at 72°C for 7 min for all molecules. PCR-amplified products (10 μl) were electrophoresed through a 1,8% agarose gel (Ambion Inc., Austin, Tx, USA) containing 0,5 μg/ml of ethidium bromide and compared with DNA reference markers. Products were visualized by ultraviolet illuminations. Polaroid photographs with ultraviolet exposure were taken with a 665 Polaroid film. Bands were analyzed with the Phoretix 1D version 3.0.

### Flow cytometric analysis

Experiments to determine ICAM-1 and COX-2 expression on fibroblasts and LFA-1, CD3, CD28 and CD69 expression on lymphocytes were carried out on cells isolated and co-cultured as described before. After 36 hours of co-colture cells were lightly trypsinized, washed and resuspended in PBS with 0.1% BSA. The cells were incubated with primary antibodies, anti-LFA-1 mAb (Dako Italia, Milan, Italy), anti-CD3 and anti-CD69 mAbs (Beckman Coulter Italia, Milan, Italy), or anti-COX-2 policlonal Ab (Santa Cruz Biotechnology Inc., Santa Cruz, CA, USA) for 60 min at room temperature. Following washing, the secondary antibody, fluorescein (FITC)-conjugated rabbit anti-mouse IgG, was added for 60 min at room temperature. Controls included omission of the primary antibody and incubation only with the secondary antibody. For COX-2 detection cells were in advance permeabilized with Triton 10x for 5 min at 4°C. FITC-labeled anti-ICAM-1 (Dako Italia) and PE-labeled anti-CD28 (BD Pharmingen Italia, Milan, Italy) were also used. Samples were analyzed using a Coulter Epics Elite ESP flow cytometer (Coulter Corporation, Miami, FL, USA). Fibroblasts and lymphocytes were gated on the basis of forward and side scatter profile. Intracellular staining of cytokines was performed using a method originally developed by Laskay and Anderson [19] and recently modified by Assenmacher et al. [20]. Briefly, Brefeldin A at 10 μg/mL (Sigma-Aldrich Co., St Louis, MO, USA), was added to cultures and cocultures CD4+ T cells and fibroblasts described above, for the final 5 hours of our experimental setup. Cells were then harvested and washed once in PBS. Freshly prepared formaldehyde solution (2% in PBS) was added to the cell pellet. Cells were vigorously resuspended and fixed for 20 minutes at room temperature. After washing in saponin buffer (0.5% saponin and 1% BSA in PBS) (Sigma-Aldrich Co., St Louis, MO, USA) the cells were stained, for 1 hour in the dark, in 100 mcl of saponin buffer containing FITC- or PE-conjugated anti-cytokine antibody at the following concentrations: anti-IL10-PE (2.5 μg/ml), anti-IFNγ-PE (2.5 μg/ml), anti-TNFalpha-PE (2.5 μg/ml), anti-IL4-FITC (5 μg/ml) (Caltag Laboratories, Burlinghame, CA, USA). Thereafter, the cells were washed three times with saponin buffer, once with PBS and analyzed by flow cytometry. Gating was always restricted on T cells. Therefore, all depicted data are given in percent of CD4+ T cells. Control stainings with PE- or FITC-coupled isotype-matched antibody were performed in preliminary experiment and never stained >0.3% of CD4+ T cells.

At least 10,000 forward and side scatter gated events were collected per specimen. Cells were excited at 488 nm and the fluorescence was monitored at 525 nm. Fluorescences were collected using logarithmic amplification.

### Lymphocytes proliferation assay

After 36 hours co-culture protocol CD4+ lymphocytes were harvested and plated at a density of 2.5 × 10^5 ^cells in 24 well plates in supplemented RPMI with 2,5 mcg/ml Concanavalin A (Con A, Sigma-Aldrich Co.) and incubated for 72 hours at 37°C in a 5% CO2 atmosphere, lymphocytes co-cultured with fibroblasts were very gently harvested with warm PBS to detach them from the fibroblasts monolayer, CD4+ T cells harvested this way, had always more than 95% of purity as assessed by differential cell counts and by flow cytometry. Thereafter medium was removed, cells were incubated with fresh medium containing 3-(4,5-dimethylthiazol-2-yl)-2,5-diphenyltetrazolium bromide (MTT) (Sigma-Aldrich Co.) at a final concentration of 0.9 mg/ml for 2 h at 37°C. The solubilization solution, containing acidified isopropanol and 20% SDS, was added and left for 20 min in order to extract the produced formazan which was then evaluated in a plate reader (absorbance = 560 nm).

### Statistical analysis

Statistical comparisons of the levels of expression of ICAM-1, COX-2, LFA-1, CD3, CD28, CD69, IL10 and TNF-alpha in all different experimental conditions were performed using a two-way analysis of variance (ANOVA) followed by the Newman-Keuls test for comparisons of specific means, the same tests were used to assess differences among T cells proliferative responses to Con A. A p value of less than 0.05 was considered significant. Results are expressed as mean ± SE.

## Results

**Figure 1:**
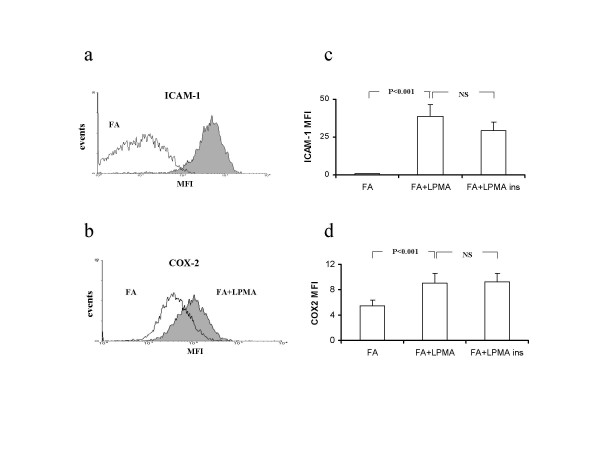
Representative flow cytometry histograms of ICAM-1 (a) and COX-2 (b) in fibroblast alone (FA), fibroblasts co-cultured with PMA-stimulated lymphocytes (FA+LPMA). ICAM-1 (c) and COX-2 (d) expression in fibroblasts, fibroblasts co-cultured with PMA-stimulated lymphocytes, fibroblasts co-cultured with PMA-stimulated lymphocytes in the presence of a semipermeable membrane (FA+LPMA ins.) Data represent means ± SE of seven independent experiments in which seven different cells lines were used.

**Figure 2:**
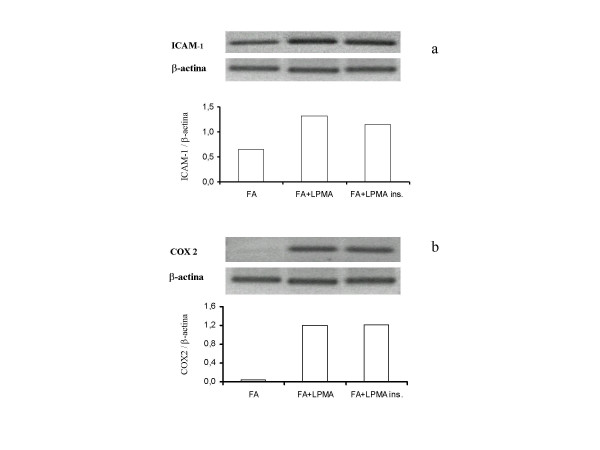
Levels of mRNA for ICAM-1 (a) and COX-2 (b) in fibroblasts (FA), fibroblasts co-cultured with PMA-stimulated lymphocytes (FA+LPMA), fibroblasts co-cultured with PMA-stimulated lymphocytes in the presence of a semipermeable membrane (FA+LPMA ins.). In the upper panels, modifications in the appearance of a 367-bp (ICAM-1) and a 305-bp (COX-2) band are compared with that of a β-actin. In the lower panels, the densitometric analysis is shown. Data are from one experiment representative of three.

Cultured lung fibroblasts, under basal conditions, expressed both ICAM-1 and COX-2 as measured by mean fluorescence intensity (MFI) of positive cells at flow cytometric analysis. Exposure of fibroblasts to resting lymphocytes produced an increased expression of both ICAM-1 (from 0.7 ± 0.4 to 2.5 ± 1.4) and COX-2 (from 5.4 ± 0.9 to 6.8 ± 1.1) that however did not yield statistical significance. In contrast, co-incubation of fibroblasts with activated lymphocytes determined a pronounced increase of the expression of both ICAM-1 and COX-2 MFI to 38.6 ± 7.8 (P < 0.001) and 9.1 ± 1.5 (P < 0.001), (Fig. 1a–d). This effect was fully preserved when the two cell types were maintained physically separated by a semi-permeable membrane. The ability of activated lymphocytes to affect ICAM-1 and COX-2 expression was likely exerted at the transcriptional level as suggested by RT-PCR that revealed increased ICAM-1 and COX-2 transcripts in fibroblasts incubated with activated lymphocytes for 36 h (Fig 2a,b). Again, the increased expression was maintained in the presence of a separating semi-permeable membrane.

**Figure 3:**
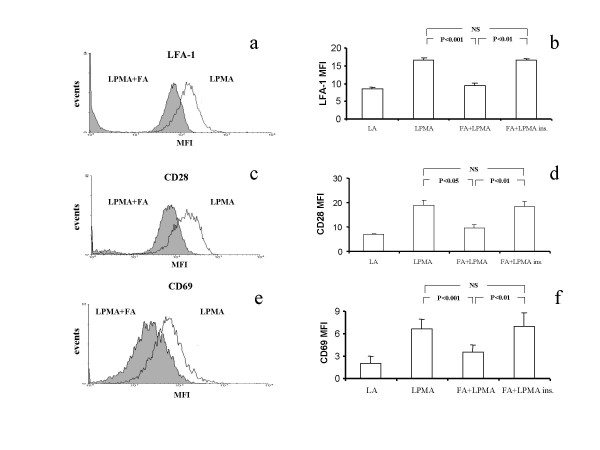
Representative flow cytometry histograms of LFA-1 (a), CD28 (c) and CD69 (e) in PMA-stimulated lymphocytes (LPMA), PMA-stimulated lymphocytes co-cultured with fibroblasts (LPMA+FA). LFA-1 (b), CD28 (d) and CD69 (f) expression in resting lymphocytes (LA), PMA-stimulated lymphocytes, PMA-stimulated lymphocytes co-cultured with fibroblasts and PMA-stimulated lymphocytes co-cultured with fibroblasts in the presence of a semipermeable membrane (LPMA+FA ins.). Data represent means ± SE of seven independent experiments in which seven different cells lines were used.

**Figure 4:**
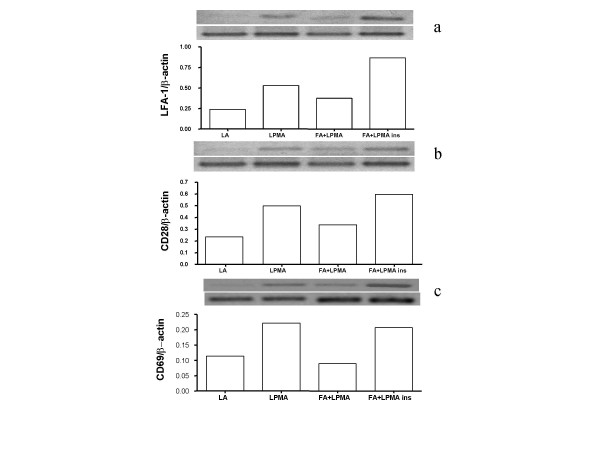
Levels of mRNA for LFA-1 (a), CD28 (b) and CD69 (c) in resting lymphocytes (LA), PMA-stimulated lymphocytes (LPMA), PMA-stimulated lymphocytes co-cultured with fibroblasts (LPMA+FA) and PMA-stimulated lymphocytes co-cultured with fibroblasts in the presence of a semipermeable membrane (LPMA+FA ins.). In the upper panels, modifications in the appearance of a 337-bp (LFA-1), a 304-bp (CD28) and a 451-bp (CD69) band are compared with that of a β-actin. In the lower panels, the densitometric analysis is shown. Data are from one experiment representative of three.

Expression of LFA-1, CD28 and CD69 in lymphocytes was markedly increased by exposure to 10 ng/ml PMA for 6 h, from 8.4 ± 0.5 for LFA-1, 6.9 ± 0.4 for CD28 and 2.6 ± 0.6 for CD69 to 16.6 ± 0.7 (P < 0.001), 18.9 ± 1.9 (P < 0.001) and 6.6 ± 1.3 (P < 0.01), respectively (Fig. 3a–f). However, co-incubation of activated lymphocytes with fibroblasts, significantly reduced the expression of LFA-1, CD28 and CD69 to 9.4 ± 0.7 for LFA-1 (P < 0.001), 9.4 ± 1.4 for CD28 (P < 0.001) and 3.5 ± 1.0 (P < 0.05) for CD69, an effect that required the physical contact between the two cell types, as suggested by the fact that it was not present any more when a semi-permeable membrane was applied (Fig. 3b,d,f). In this condition, MFI was 16.5 ± 0.6 for LFA-1, 18.4 ± 1.9 for CD28 and 7.1 ± 1.8 for CD69, all these values being statistically increased compared to cells cultured without the membrane (P < 0.01) and not significantly different to those observed in activated lymphocytes cultured in absence of fibroblasts. RT-PCR revealed that the reduced expression of LFA-1, CD28 and CD69 was related to their decreased transcription (Fig. 4a–c) and, similarly to what observed with protein expression, the reducing effect of fibroblasts was prevented by the presence of a membrane between the two cell types (Fig. 4a–c).

**Figure 5:**
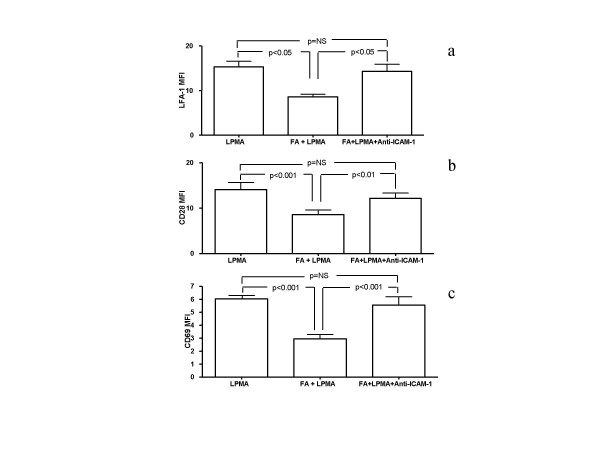
LFA-1 (a), CD28 (b) and CD69 (c) expression in PMA-stimulated lymphocytes (LPMA), PMA-stimulated lymphocytes co-cultured with fibroblasts (LPMA+FA) and PMA-stimulated lymphocytes co-cultured with fibroblasts in the presence of an anti ICAM-1 blocking antibody (LPMA+FA+anti ICAM-1). Data represent means ± SE of four independent experiments.

To ascertain whether the reduced protein expression could be ascribed to activation of ICAM-1 on lymphocyte surface, the same experiment was performed in the presence of a blocking anti-ICAM-1 antibody. Under these conditions, lymphocyte expression of LFA-1, CD28 and CD69 was fully restored, being statistically increased compared to cells cultured with fibroblasts and not significantly different to those observed in activated lymphocytes (Fig. 5a–c).

**Figure 6:**
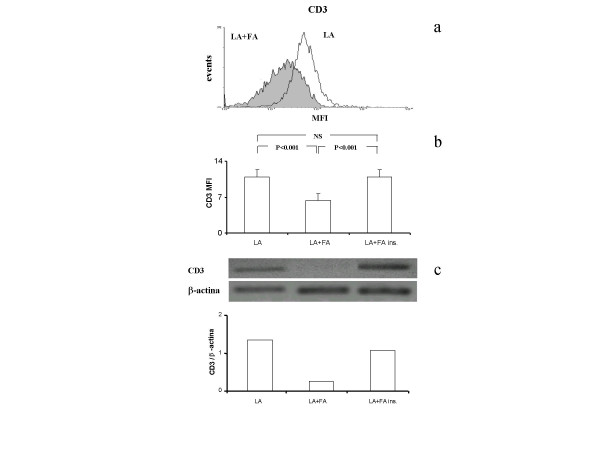
(a) Representative flow cytometry histogram of CD3 in resting lymphocytes (LA) and in resting lymphocytes co-cultured with fibroblasts (LA+FA). (b) CD3 expression in resting lymphocytes, resting lymphocytes co-cultured with fibroblasts and resting lymphocytes co-cultured with fibroblasts in the presence of a semipermeable membrane (LA+FA ins.). Data represent means ± SE of seven independent experiments in which seven different cells lines were used. (c) Levels of mRNA for CD3 in resting lymphocytes, resting lymphocytes co-cultured with fibroblasts and resting lymphocytes co-cultured with fibroblasts in the presence of a semipermeable membrane. In the upper panel, modifications in the appearance of a 496-bp (CD3) band are compared with that of a β-actin. In the lower panel, the densitometric analysis is shown. Data are from one experiment representative of three.

Expression of CD3 was evaluated in resting lymphocytes as activation by PMA did not significantly modify expression of the protein. Co-incubation of resting lymphocytes with fibroblasts significantly reduced CD3 expression both at translational (Fig. 6a,b) and transcriptional (Fig. 6c) level, an effect that was completely prevented in the presence of a separating membrane. Indeed, CD3 MFI was reduced from 11.9 ± 1.4 to 6.4 ± 1.1 (P < 0.001), while in experiments performed with a separating membrane, CD3 MFI was restored to (11.0 ± 0.9) (P < 0.001).

**Figure 7:**
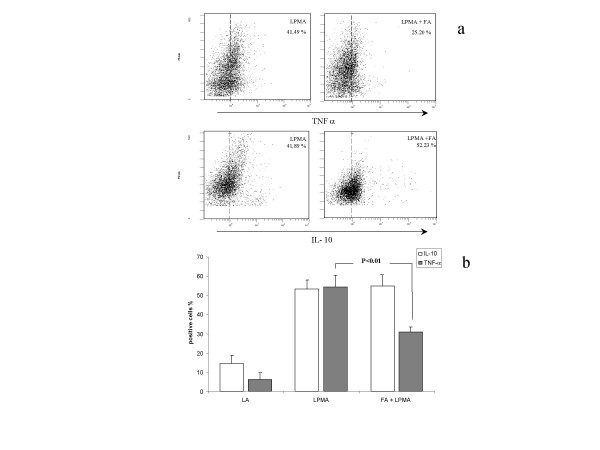
(a) Representative flow cytometry dot plots of TNF-α and IL-10 positive PMA-stimulated lymphocytes (LPMA) and PMA-stimulated lymphocytes co-cultured with fibroblasts (LPMA+FA). (b) TNF-α and IL-10 positive cells, expressed as percentage, among resting lymphocytes (LA), PMA-stimulated lymphocytes and PMA-stimulated lymphocytes co-cultured with fibroblasts. Data represent means ± SE of 4 independent experiments.

**Figure 8:**
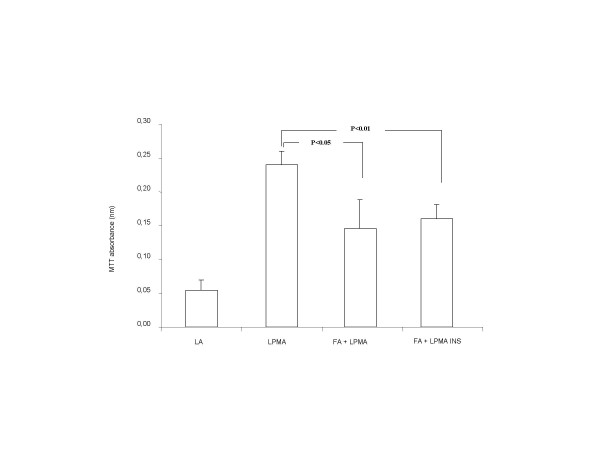
Proliferative responses of resting lymphocytes (LA), PMA-stimulated lymphocytes (LPMA), PMA-stimulated lymphocytes co-cultured with fibroblasts (LPMA+FA) and PMA-stimulated lymphocytes co-cultured with fibroblasts in the presence of a semipermeable membrane (LPMA+FA ins.), measured by means of MTT, after 72 hours culture in the presence of 2,5 μg/ml concanavalin A. Data represent means ± SE of 6 independent experiments.

Finally, in order to evaluate whether the interaction between fibroblasts and lymphocytes could give rise to changes of the function of the latter cell population, two different approaches were used. As a first step, lymphocytes exposed to fibroblasts were tested for their intracellular production of cytokines. Attention has been focused on TNF-alpha and IL-10, two cytokines whose role in lymphocyte function has been thoroughly characterized. As expected, an increase of both TNF-alpha (from 2.5% ± 5.4 to 54.4% ± 6.12, p < 0.01) and IL-10 (from 15.8% ± 5.2 to 53.3% ± 4.6, p < 0.01) positive cells was observed following activation with PMA, (Fig. 7b). Interestingly, co-incubation with fibroblasts significantly reduced the number of TNF-alpha positive lymphocytes (from 54.4% ± 6.12 to 30.8% ± 2.8, p < 0.05), without any significant change of IL-10 positive cells (from 53.3% ± 4.6 to 54.9% ± 5.9, p = NS). Finally we tested the ability of concanavalin A to induce a proliferative response in activated lymphocytes. This proliferation was partially quenched in lymphocytes co-cultured with fibroblasts (Fig. 8). Indeed, proliferation measured as MTT absorbance was reduced from 0.24 nm ± 0.02 of activated lymphocytes to 0.15 nm ± 0.04, p < 0.05, an effect fully maintained when a semi-permeable membrane was applied (0.16 nm ± 0.02, p < 0.01).

## Discussion

A great deal of evidence is today available showing that resident cells such as fibroblasts, through the release of soluble signals and/or direct interactions with other cells, may serve as potential regulators of the local inflammatory response [21]. Based on our previous findings, human lung fibroblasts, through the modulation of some monocyte activities, may also participate in the control of the immune response [1]. We have shown that normal human lung fibroblasts are able to interact with monocytes, driving the release of cytokines, whose role is crucial in the regulation of the immune response, i.e. they strongly stimulate interleukin 10 (IL-10) production by LPS-activated monocytes and inhibit interleukin 12 (IL-12). The increase of IL-10 production, induced by fibroblasts, is able, in an autocrine way, to downregulate the expression of human leukocyte-associated antigen-DR (HLA-DR) as well as the expression of CD40 on monocytes, potentially affecting the antigen presenting capacity of these cells as well as their costimulatory function. In addition, we have also shown that an impairment of fibroblast functions, as observed in fibrotic fibroblasts, may lead to a reduced capability of these cells to modulate monocyte activity [1].

In view of the above mentioned experimental evidence we set out to determine whether the interaction between normal human lung fibroblasts and immune cells such as T-lymphocytes could lead to a functionally significant response by these cells.

Several lung diseases, including hypersensitivity pneumonitis, sarcoidosis and bronchial asthma are in fact characterized by an involvement of T-lymphocytes and a subsequent impairment of the immune and inflammatory response [3,22-24]. Another element, common to these diseases, is represented by the possibility that the inflammatory state may ultimately lead to fibroblast activation and tissue fibrosis. Indeed, interstitial lung diseases are marked by fibrosis and also bronchial asthma is characterized by an extensive remodeling of the bronchial wall due to fibroblast activation and collagen deposition [25-27].

Our results indicate that lung fibroblasts and T-lymphocytes mutually interact. Activated lymphocytes induce COX-2 mRNA accumulation and protein expression and dramatically increase both transcription and expression of ICAM-1 in normal human lung fibroblasts. Fibroblasts, in turn, induce a significant reduction of transcription and protein expression of CD69, a marker of early T activation, and LFA-1, CD3 and CD28, all molecules involved in T-lymphocyte activation and costimulation. Moreover, TNF-alpha a typical proinflammatory cytokine [28], was significantly inhibited by fibroblasts, whereas IL-10, commonly considered as a regulatory cytokine [29] was not affected by fibroblasts. Finally, we have demonstrated that lymphocytes co-cultured with fibroblasts show a significantly reduced proliferative response to a mitogenic stimulus.

The enhanced expression of both COX-2 and ICAM-1 on fibroblasts, induced by activated lymphocytes, was not affected by the presence of a semi-permeable membrane separating fibroblasts and lymphocytes, suggesting that the T cell-induced fibroblast activation is likely mediated by soluble factors produced by lymphocytes. The increased expression of COX-2 induced by T cells on fibroblasts is of great interest considering that a large number of studies depicts COX-2 and its products, prostaglandins, as a major pathway occurring in the lung during the control and self-limitation of the inflammatory and reparative process [30]. As regard as the increased expression of ICAM-1 on fibroblasts cocultured with lymphocytes, it has already been shown that various cytokines produced by mononuclear cells enhance adhesiveness of fibroblasts for T cells, through up-regulation of fibroblast ICAM-1 expression, promoting T cell retention, positioning and accumulation in the tissues [31,32]. This observation suggests that T cells, once migrated into the tissue, facilitate their own retention by boosting local fibroblast adhesive properties. This event is commonly considered important for the persistence of inflammation and blockade of the interaction between resident cells and T cells may eventually down-regulate inflammation, representing an ideal target for disrupting immune-non-immune cell interaction. To this regard, in different animal experimental models attenuation of inflammation and reduction in collagen deposition have been described when neutralizing antibodies against adhesion molecules are used [33,34]. However, in an ICAM-1 knockout mice, bleomycin has been reported to induce a more severe pulmonary fibrosis compared to their wild-type counterparts [35]. These results confirm the importance of adhesion molecules for the recruitment and accumulation of inflammatory cells into the tissues and, most importantly, suggest their fundamental role in cell-to-cell communication and inflammation control. Indeed, our findings demonstrate that the adhesion and subsequent interaction between lymphocytes and fibroblasts have an important role in down-regulating T cell activation and likely in dampening the activation state of these cells that characterizes some lung diseases.

With regard to this we have shown that the inhibitory effect exerted by fibroblasts on T lymphocytes activity is evident at multiple levels: surface markers expression, cytokines production and proliferation.

We have shown that the inhibitory effect exerted by fibroblasts on the expression of LFA-1, CD3, CD28 and CD69 on lymphocytes is due to a direct interaction since the inhibitory effect is abolished when a membrane is placed between fibroblasts and lymphocytes. Considering that ICAM-1 expression on fibroblasts makes these cells capable to physically interact with beta 2 integrin positive inflammatory leukocytes and its expression is increased by activated lymphocytes, we hypothesized that the interaction between activated T cells and fibroblasts was ICAM-1 mediated. Indeed, co-culture experiments performed preincubating fibroblasts with a neutralizing anti-ICAM-1 antibody abolished their capacity to reduce LFA-1, CD28 and CD69 expression on activated lymphocytes suggesting that a cognate interaction, LFA-1-ICAM-1 mediated, is requested for the inhibitory effect of fibroblasts on lymphocytes. Similarly, the inhibitory effect induced by fibroblasts on T cell CD3 expression has to be ascribed to a direct interaction, as demonstrated by the disappearance of the inhibitory activity in the presence of a membrane physically separating fibroblasts and lymphocytes. The inhibition of CD3 expression as well as the simultaneous reduction of LFA-1, CD28, CD69 that we have described on lymphocytes cultured with fibroblasts may have important effects on the evolution of the immune response. A full T cell activation depends, in fact, (i) on the interaction of the T cell receptor (TCR)-CD3 complex with the antigen and (ii) on a second signal delivered through the costimulatory molecule CD28, necessary for the complete activation [36,37]. The binding of CD28 and its ligands B7-1 and B7-2 lowers the threshold number of TCR molecules that need to be engaged to initiate T cell activation, protects against the induction of T cell anergy, promotes proliferation, cytokine production and T cell survival [37,38]. In addition, the interaction between T cell-mounted LFA-1 and its ligand ICAM-1 functions not only as an adhesive interaction, but may also deliver a further costimulatory signal able to activate T cells and is considered important for T cell- antigen presenting cell (APC) interaction [39]. Several data suggest that these molecules are crucial in the pathogenesis of a number of lung diseases. Specifically, an influx of activated T-cells characterizes hypersensitivity pneumonitis and the blockade of T-cell costimulation inhibits lung inflammation in a murine model of this disease [23,40]. In pulmonary sarcodosis, a number of reports have emphasized the role of pulmonary lymphocytes and described phenotypic and functional abnormalities of T-cells [22,41] whereas there is evidence showing that T-lymphocytes may also have a role in the fibrotic process, although much remains to be defined. In this regard Okazaky et al. [42] have recently shown that CD28-mediated T-cell costimulation plays a critical role in the development of inflammation and fibrosis in bleomycin-treated mice. On the contrary, the administration of antibodies to LFA-1 leads to a significant reduction in collagen deposition in lung tissue both in experimental hypersensitivity pneumonitis [43] and pulmonary fibrosis [33]. Furthermore, allergen-induced production of T-cell derived cytokines in asthma requires the interaction between costimulatory molecules and points to the CD28-B7 pathway as being important to the inflammation distinctive of the disease [44].

To ascertain whether the phenotypical changes above described were accompanied by a certain degree of functional alteration we studied cytokine production and proliferation of co-cultured T lymphocytes.

Specifically we evaluated the production of TNF-alpha and IL-10 by lymphocytes co-cultured with fibroblasts. TNF-alpha, is a proinflammatory cytokine with many biologic properties thought to be critical in the development of pulmonary fibrosis [28]. Whereas, IL-10 is a regulatory cytokine, that suppresses the production of several proinflammatory cytokines [29], likely through the inhibition of NF-kB activation [45].

According to the immunoregulatory role that we have suggested for fibroblasts, these cells inhibit in T lymphocytes the production of the proinflammatory cytokine TNF-alpha whereas the release of the anti-inflammatory cytokine IL-10 is not affected. Moreover, T-lymphocytes co-cultured with fibroblasts show a significantly reduced proliferative response when exposed to a well known mitogenic stimulus such as concanavalin A [46], underlining the immunoregulatory role exerted by fibroblasts on activated t-lymphocytes.

Thus, several lines of evidence indicate that T-lymphocytes, through the production of soluble signals and direct interactions with other cells orchestrate the immune as well as the inflammatory response and may consequently be involved in the pathogenesis of several lung diseases. In this study, we have shown that CD3, CD28, CD69 and LFA-1, some of the main signals identifying T-cell activation, and taking active part in the immune response, besides TNF-alpha production, are down-regulated by human normal lung fibroblasts, affecting the capacity of these cells to proliferate in response to a non specific stimulus and potentially altering the interaction with antigen presenting cells. This study confirms and expands the concept that tissue resident cells such as fibroblasts do have a role in the regulation of some aspects of the immune response acting as a highly effective local control system of the effector functions of cells recruited into sites of inflammation. An impairment of this modulatory attitude can make the difference between acute self-limited inflammation followed by normal repair as compared to chronic unquenchable inflammation leading to loss of tissue architecture and fibrosis.
